# Application of Gurson–Tvergaard–Needleman Constitutive Model to the Tensile Behavior of Reinforcing Bars with Corrosion Pits

**DOI:** 10.1371/journal.pone.0054368

**Published:** 2013-01-14

**Authors:** Yidong Xu, Chunxiang Qian

**Affiliations:** 1 School of Materials Science and Engineering, Southeast University, Nanjing, Jiangsu, P. R. China; 2 Research Center of Green Building Materials and Waste Resources Reuse, Ningbo Institute of Technology of Zhejiang University, Ningbo, Zhejiang, P. R. China; Dalhousie University, Canada

## Abstract

Based on meso-damage mechanics and finite element analysis, the aim of this paper is to describe the feasibility of the Gurson–Tvergaard–Needleman (GTN) constitutive model in describing the tensile behavior of corroded reinforcing bars. The orthogonal test results showed that different fracture pattern and the related damage evolution process can be simulated by choosing different material parameters of GTN constitutive model. Compared with failure parameters, the two constitutive parameters are significant factors affecting the tensile strength. Both the nominal yield and ultimate tensile strength decrease markedly with the increase of constitutive parameters. Combining with the latest data and trial-and-error method, the suitable material parameters of GTN constitutive model were adopted to simulate the tensile behavior of corroded reinforcing bars in concrete under carbonation environment attack. The numerical predictions can not only agree very well with experimental measurements, but also simplify the finite element modeling process.

## Introduction

Structure deterioration induced by corrosion of reinforcing bars is one of the major problems in civil engineering. The corrosion of reinforced concrete structures can not only lead to the cracking of the concrete cover [1]–[5], but also the serious damage of reinforcing bars [6]–[12]. Therefore, investigation of the deterioration of mechanical properties of corroded steel bars is crucial for predicting the serviceability and durability of reinforced concrete structures. Empirical formulas have been proposed to evaluate the yield and ultimate strengths of corroded reinforcing bars. The mathematical models of stress-strain relationship for corroded rebars in different environment condition have also been established. However, these observed macroscopic experimental phenomena cannot reflect the relationship between the macro- and meso-material characteristics, in which the former is related to the mechanical weakening of the material and the latter is associated with the large number of randomly distributed defects of irregular shapes, sizes and orientations.

For reinforced concrete structures the corrosion of reinforcing bars is often caused due to chloride attack and carbonation, which is a “localized” pitting corrosion. Many researchers have investigated the stress concentration effect of single pits with various pit depth and diameter [13]–[16]. The detailed finite element analyses have been conducted to evaluate the stress and strain distribution around a pit in a cylindrical specimen [17]. However, explicit modeling of the voids is not usually practical because of the large difference in the structure and meso-void scales [18]. From the mechanics point of view, it is much convenient to use the concept of damage variables describing the corrosion characteristic and corresponding damage evolution. The key in developing such a relationship is the establishment of damage constitutive model by introducing meso-defect into macro volume element, and the related macroscopic constitutive relation can be predicted.

In this paper, the porous metal plasticity model, GTN constitutive model, is introduced to describe the tensile behavior of corroded reinforcing bars. The effect of different GTN constitutive model parameters on the numerical simulation results of material tensile property is conducted by using orthogonal test method. The GTN constitutive model with calibrated parameters is applied to predict the tensile behavior of reinforcing bars with various corrosion degrees, and the numerical predictions agree very well with experimental measurements.

## Materials and Methods

### Materials

**Figure 1:**
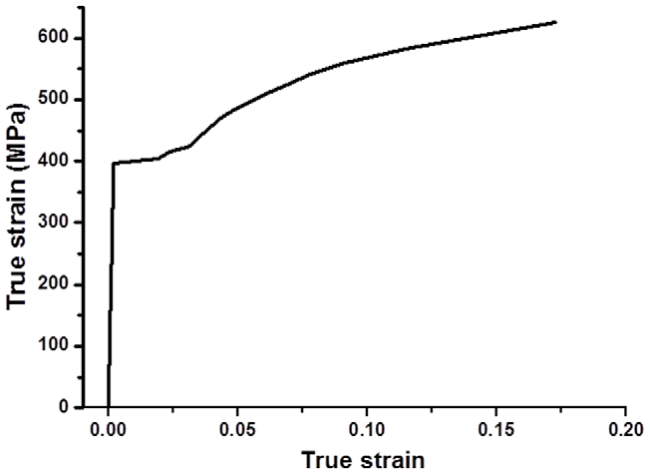
True stress-strain curve of uncorroded reinforcing bar.

The designed concrete compressive strength is 30 MPa. The mixture of the concrete is shown in Table 1. Hot rolled plain steel bar (with nominal diameter of 12 mm) according to ISO Standards 6935-1 was used. The true stress-strain curve of uncorroded reinforcing bar is shown in Figure 1. The size of the reinforced concrete specimens is 100 mm*100 mm*400 mm.

**Table 1:** Concrete proportion (kg/m^3^).

Type 42.5 Portland cement	Water	Fine aggregate	Coarse aggregate
365	192	730	1095

### Test Methods

**Figure 2:**
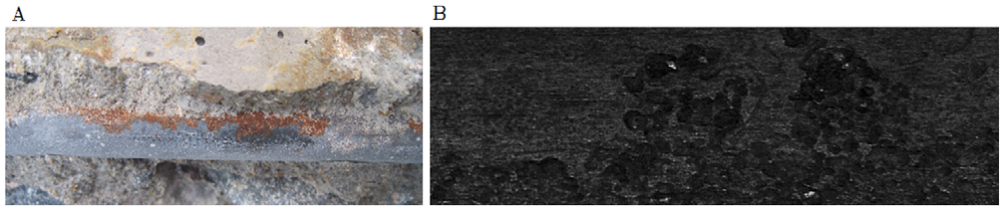
Reinforced concrete specimen under carbonation environment attack. (A) Corroded reinforced concrete specimen, (B) Pitting corrosion morphology.

After curing in a fog room (20±2°C, 95% RH) for 28 days, the reinforced concrete specimens were placed inside a carbonation tank to allow the corrosion of reinforcing bars in concrete under carbonation environment attack. After being removed from the concrete, reinforcing bars (marked as R1–R4) were washed by using a de-rusting solution to remove corrosion products. The de-rusting solution was prepared by mixing 3 parts by mass of hexamethylene tetramine (analytical reagent) into 97 parts diluted hydrochloric acid. The corrosion mass loss ratio of rebar specimen then was calculated. Figure 2 shows the corroded reinforced concrete specimen and typical pitting corrosion morphology of reinforcing bar. These corrosion morphologies have generally irregular shapes, sizes and orientations. Some of local corrosion morphologies are overlapped or connected together.

**Figure 3:**
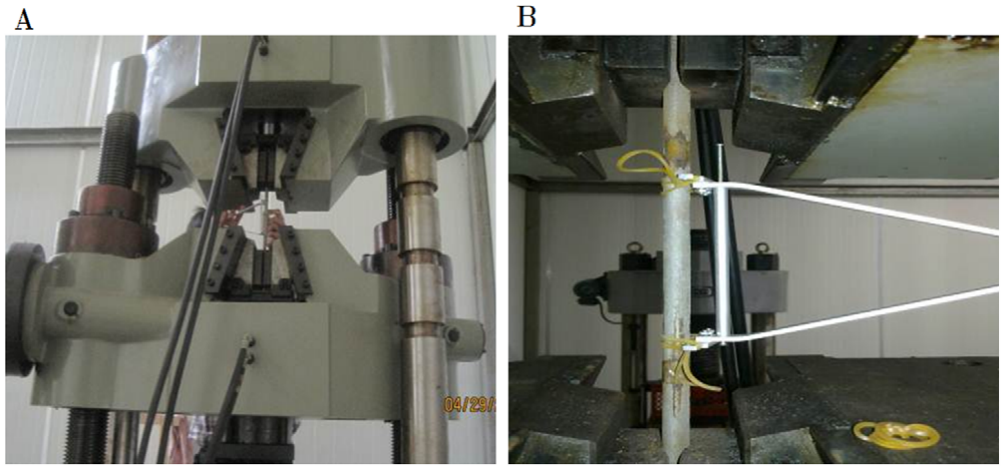
Equipment for tensile test. (A) Electro-hydraulic servo testing machine, (B) Electronic extensometer.

Tensile test was performed for the rebar specimen using standard strength test procedure according to ISO Standards 6892∶1998 to obtain the nominal yield and ultimate strengths of the rebar. In the tensile test an electro-hydraulic servo testing machine was used and an electronic extensometer with gauge length of 100 mm was installed in the corrosion region to obtain the stress-strain curve of reinforcing bar, as is shown in Figure 3.

### GTN constitutive model

For most engineering alloys, ductile fracture often comes after the nucleation, growth and coalescence of microvoids [19]. By using the homogenization method of mesomechanics, Gurson derived the pressure dependent yield function from an isolated spherical void in a continuum media to describe the constitutive response of the metal [20]. The void volume fraction 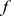 is chosen as damage parameter. Tvergaard and Needleman have subsequently introduced new material parameters to model the complete loss of load-carrying capacity at a realistic void volume fraction. The modified yield function 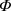 is the Gurson –Tvergaard–Needleman (GTN) model, as is shown in Eq.(1).
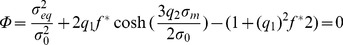
(1)where 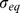 is von Mises equivalent stress, 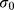 is the microscopic yield stress of the undamaged matrix material, 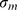 is the mean normal stress, 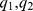 are constitutive parameters introduced by Tvergaard to modify the original Gurson model [21].


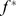
 is a function of the void volume fraction 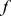, which accounts for rapid loss of stress carrying capacity due to void coalescence. This function is defined by Eq.(2).
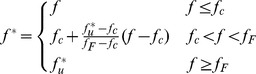
(2)where 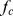 is the critical void fraction at void coalescence. 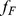 is the void volume fraction at fracture. 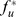 is the ultimate value of the damage parameter defined by 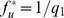.

In general, both the growth of existing voids and nucleation of new voids contribute to the increase of total void volume fraction. The evolution equation for void volume fraction can be depicted in a rate form, as is shown in Eq.(3).
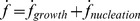
(3)


The matrix material is assumed to be plastically incompressible. The growth rate of existing voids is given by
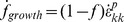
(4)where 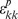 is the plastic hydrostatic strain.

The nucleation of voids is a very complex physical process. A normal distribution of void nucleation with respect to the plastic strain of the matrix material 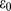 is proposed by Chu and Needleman [22].
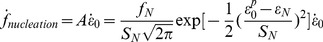
(5)where 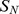 and 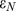 are the standard deviation and the mean value of the distribution of the plastic strain, which can be arbitrary fixed as 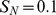 and 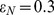. 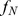 is the volume fraction of void nucleating particles, which can be evaluated by microscopical examination of the undamaged material. 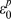 is the equivalent plastic strain of matrix material.

The GTN constitutive model can describe the influence of voids on plasticity properties of metal. If the corrosion pits of rebar is supposed to be sphere, then the corrosion mass loss ratio equals to the original void volume fraction 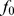. Therefore, the GTN constitutive model was introduced into finite element analysis to describe the tensile behavior of corroded reinforcing bars.

### Orthogonal array of GTN model parameters

The material parameter of GTN constitutive model can be classified into three principal families: (1) constitutive parameters, 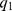 and 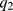; (2) void evolution parameters, 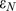 and 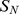; (3) failure parameters, 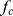 and 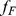. Benseddiq summarized the large mass of data available in different literatures in order to examine the validity of the choices of these parameters [23]. At present there is no unique method to determine these parameters. As is shown in Eq.(1)-Eq.(5), there are still constitutive parameters and failure parameters need to be determined. For a problem with four design variables, the orthogonal array (L_9_(3^4^)) can be used for experiment arrangement and data analysis. The selected variables and levels are shown in Table 2 and the experimental arrangement is shown in Table 3.

**Table 2:** Variables and levels for orthogonal test.

Level	Variables			
	(A) *q*_1_	(B) *q*_2_	(C) *f_c_*	(D) *f_F_*
(1)	1.5	0.5	0.1	0.2
(2)	2.5	1.0	0.15	0.25
(3)	3.5	1.5	0.2	0.3

**Table 3:** Experimental arrangement and range analysis.

Experiment No.	A		B		C		D		Strength /MPa	
	*q_1_*		*q_2_*		*f_c_*		*f_F_*		*σ_y_*	*σ_t_*
1#	A1 (1.5)		B1 (0.5)		C1 (10%)		D1 (20%)		388.47	522.33
2#	A1 (1.5)		B2 (1.0)		C2 (15%)		D2 (25%)		387.85	517.23
3#	A1 (1.5)		B3 (1.5)		C3 (20%)		D3 (30%)		386.05	507.25
4#	A2 (2.5)		B1 (0.5)		C2 (15%)		D3 (30%)		379.75	503.40
5#	A2 (2.5)		B2 (1.0)		C3 (20%)		D1 (20%)		377.40	492.40
6#	A2 (2.5)		B3 (1.5)		C1 (10%)		D2 (25%)		373.36	468.17
7#	A3 (3.5)		B1 (0.5)		C3 (20%)		D2 (25%)		369.23	484.59
8#	A3 (3.5)		B2 (1.0)		C1 (10%)		D3 (30%)		366.02	463.97
9#	A3 (3.5)		B3 (1.5)		C2 (15%)		D1 (20%)		360.47	422.80
Range analysis	*σ_y_*	*σ_t_*	*σ_y_*	*σ_t_*	*σ_y_*	*σ_t_*	*σ_y_*	*σ_t_*		
*K_1_*	387.46	515.60	379.15	503.44	375.95	484.82	375.45	479.18		
*K_2_*	376.84	487.99	377.09	491.20	376.02	481.14	376.81	490.00		
*K_3_*	365.24	457.12	373.29	466.07	377.56	494.75	377.27	491.54		
*R*	22.22	58.48	5.86	37.37	1.61	13.60	1.83	12.36		

### Finite element model

**Figure 4:**
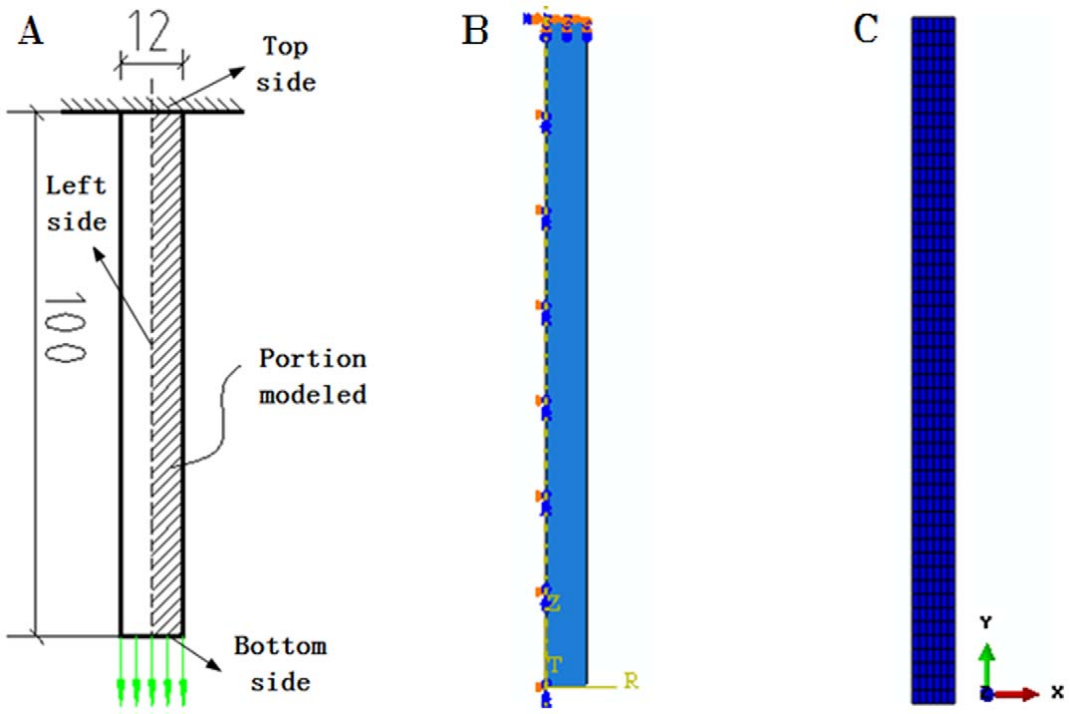
Geometry and mesh for the round tensile bar. (A) Geometry model, (B) Boundary condition, (C) Mesh dividing.

Finite element analysis was carried out using ABAQUS software. As is shown in Figure 4, the specimen diameter is 12 mm and the overall length is 100 mm. An axisymmetric cylindrical specimen was modeled in the ABAQUS CAE pre-processor. The mesh was created using the axisymmetric elements CAX4R, which is 4-node bilinear axisymmetric quadrilateral elements [15]–[17]. Due to the uncertainty of fracture position, it is considered to use refined mesh in the whole model.

The fixed boundary condition was applied to the top side of the model, and the symmetry boundary condition (XSYMM, U1 = UR2 = UR3 = 0) was applied to the left side of the model. The displacement was applied to the bottom side of the model to obtain the deformations desired in the analysis step.

The GTN constitutive model was used in the analysis to characterize the porous metal plasticity behavior of the material. Young's modulus E = 200GPa and Poisson's ratio μ = 0.3. The work hardening behavior is given by Figure 1.

In our laboratory test, the corrosion mass loss ratio of rebars due to concrete carbonation is between 0.5% and 2.5%. Therefore, this paper adopts 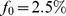 as a representative value for orthogonal numerical test. For the further validation experiments, the parameter 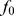 adopts the measured corrosion mass loss ratio to verify the accuracy of numerical simulation.

After the completion of computation, the calculated nominal stress strain curve could be obtained and the corresponding nominal yield strength 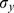 and ultimate tensile strength 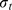 were available. During the numerical simulation of tensile test, the void volume fraction of corroded reinforcing bars will change. In order to describe the damage evolution of corroded reinforcing bars, the related void volume fraction (VVF) nephogram was also presented.

## Results and Discussion

In the range analysis of orthogonal test, *K* is the average of strength of every level and *R* scales the effect of variables on the result [24]. In order to estimate the significance of the effects of each variable, the analysis of variance technique was also employed. *S* is the sum of squares of deviations, *D_f_* is the degree of freedom, *F* shows the significance of factors' influence on the results [25]. As is shown in Table 3 and Table 4, the numerical simulation results of 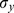 and 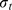 are respectively located in the following two intervals, [360MPa, 390MPa] and [420MPa, 525MPa]. According to the *R* value, the orders of influence of each variable on 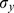 and 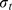 were 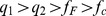, 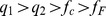, respectively. The two constitutive parameters are significant factors affecting the tensile strength. Both the nominal yield and ultimate tensile strength decrease markedly with the increase of constitutive parameters. The effect of failure parameters on the tensile strength may be negligible as compared to the constitutive parameters.

**Table 4:** Analysis of variance for strength in matrix.

Source	Index	*S*	*D_f_*	*F* a	Critical value of *F*
*q*_1_	*σ_y_*	740.847	2	142.814**	*F_0.05_* = 6.94
	*σ_t_*	5135.753		18.040**	
*q*_2_	*σ_y_*	52.959	2	10.209**	
	*σ_t_*	2177.435		7.649**	
*f_C_*	*σ_y_*	4.959	2	0.956	
	*σ_t_*	297.066		1.043	
*f_F_*	*σ_y_*	5.416	2	1.044	
	*σ_t_*	272.306		0.957	

a : ** means significantly affected.

**Figure 5:**
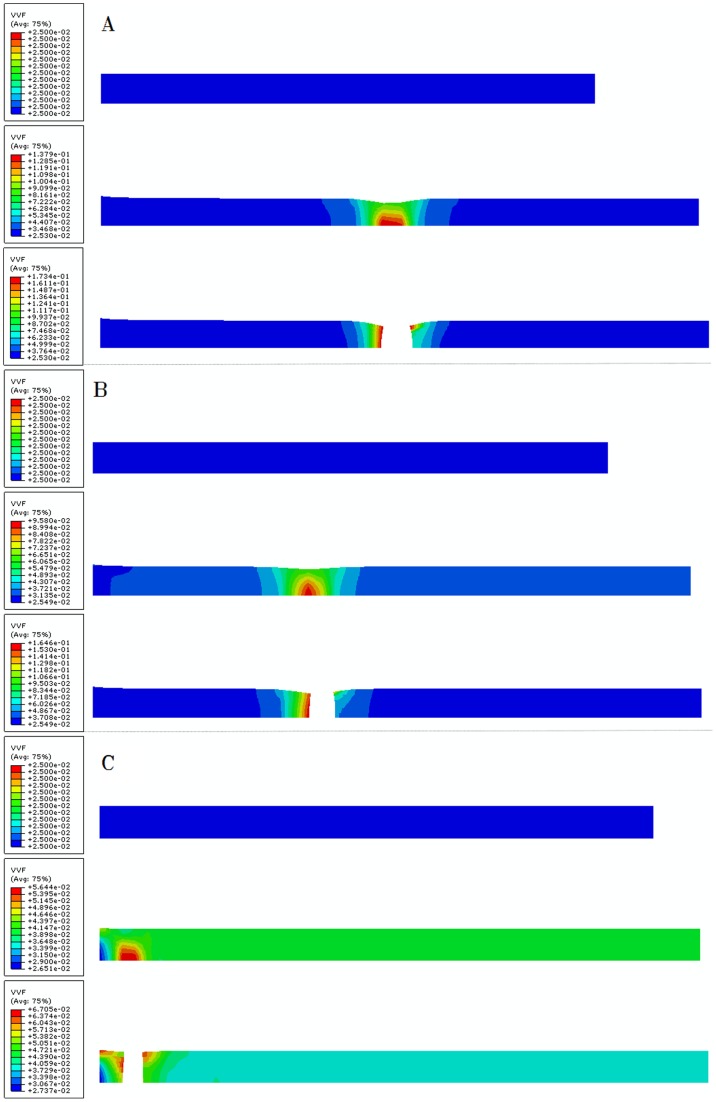
Evolution of the void volume fraction of model with different GTN material parameters. (A) 2# experiment, with typical cup-cone fracture surface, (B) 5# experiment, the necking zone moves upwards gradually, (C) 9# experiment, the necking zone is located in the top of the model and fracture surface is no longer a cup-cone pattern.

**Figure 6:**
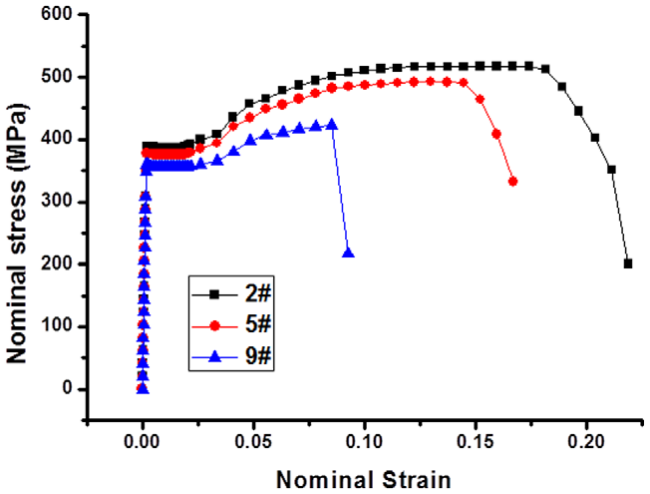
The calculated nominal stress strain curve of 2#, 5# and 9# numerical experiments. The three curves have different tensile strength and elongation.

Figure 5 shows the damage evolution process of 2#, 5# and 9# numerical experiments. For 2# experiment, the constitutive parameters are relatively small, which result in large elongation and void volume fraction of model to the failure. The necking zone is located in the middle of the model. It is observed that there exists a typical cup-cone fracture surface, which is the representation of ductile fracture. It can be seen from Figure 5 that, the breaking elongation and void volume fraction gradually decreased with the increasing of constitutive parameters. The necking zone moves upwards gradually and the fracture pattern changes from toughness to brittleness. For 9# experiment, the fracture surface is no longer a cup-cone pattern. Figure 6 shows the corresponding nominal stress-strain curves.

**Figure 7:**
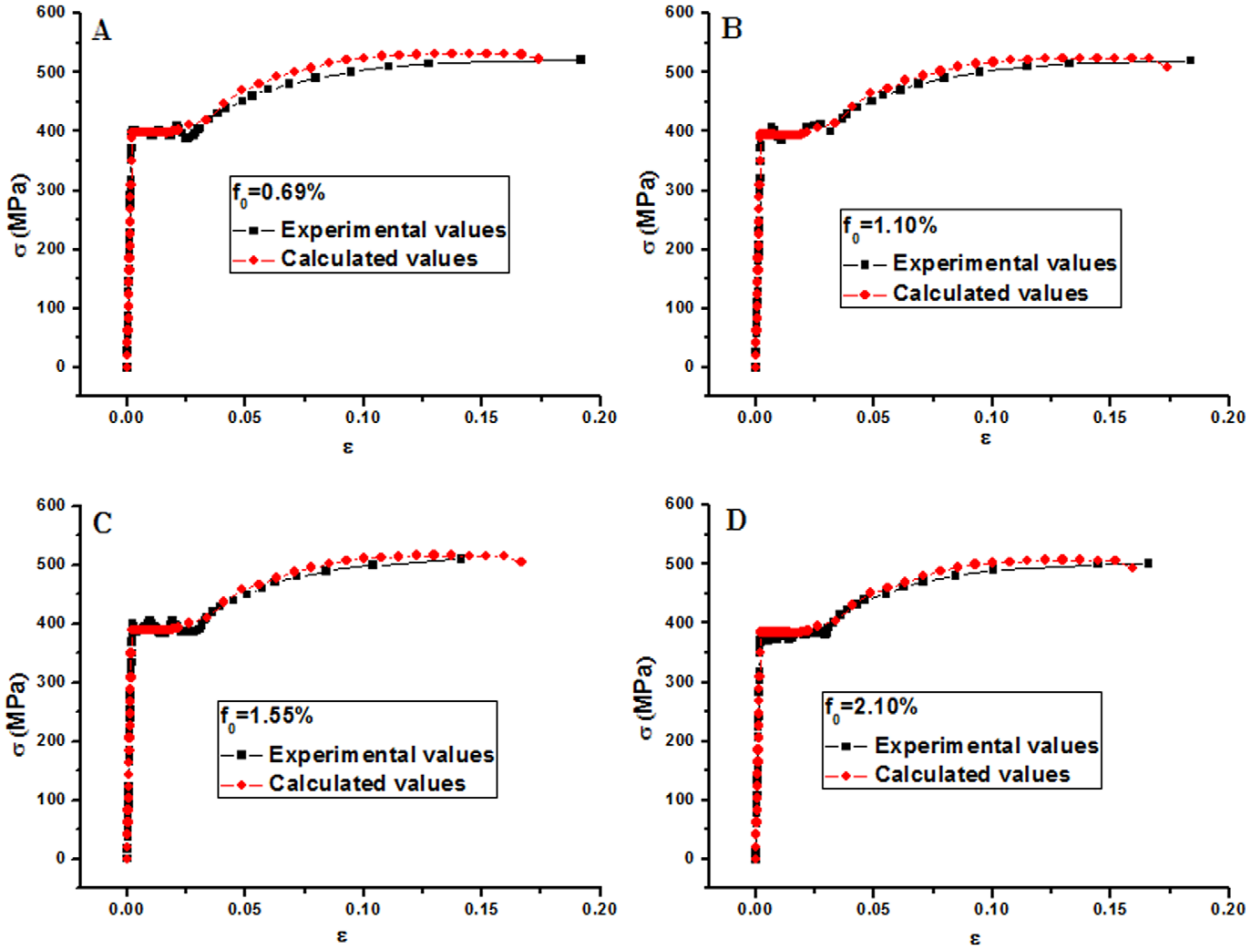
Measured nominal stress strain curves, and model predictions for corroded reinforcing bars in concrete under carbonation environment attack. (A) R1 specimen, (B) R2 specimen, (C) R3 specimen, (D) R4 specimen.

The orthogonal test results show that the tensile behavior of corroded bars can be simulated by choosing rational material parameters. Combining with the latest data from Benseddiq [23] and trial-and-error method, the following material parameters for GTN constitutive model, 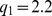, 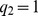, 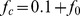, 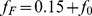, were adopted to simulate the tensile behavior of corroded reinforcing bars in concrete under carbonation environment attack. As is demonstrated in Figure 7, the numerical predictions agree very well with experimental measurements.

## Conclusions

The GTN constitutive model is introduced in finite element analysis to describe the tensile behavior of corroded reinforcing bars. By choosing different material parameters of GTN constitutive model, different fracture pattern and the related damage evolution process can be simulated. The results of orthogonal test indicate that the two constitutive parameters are significant factors affecting the tensile strength. Adopting the GTN constitutive model with calibrated parameters can not only predict the tensile behavior of reinforcing bars with various corrosion degrees accurately, but also simplify the finite element modeling process.
